# The Corneal Basement Membranes and Stromal Fibrosis

**DOI:** 10.1167/iovs.18-24428

**Published:** 2018-08

**Authors:** Carla S. Medeiros, Gustavo K. Marino, Marcony R. Santhiago, Steven E. Wilson

**Affiliations:** 1 The Cole Eye Institute, The Cleveland Clinic, Cleveland, Ohio, United States; 2 Department of Ophthalmology at University of Sao Paulo, Sao Paulo, Brazil; 3 Department of Ophthalmology at Federal University of Rio de Janeiro, Rio de Janeiro, Brazil

**Keywords:** epithelial basement membrane, scarring, myofibroblasts, Descemet's membrane, transforming growth factor beta

## Abstract

**Purpose:**

The purpose of this review was to provide detailed insights into the pathophysiology of myofibroblast-mediated fibrosis (scarring or late haze) after corneal injury, surgery, or infection.

**Method:**

Literature review.

**Results:**

The epithelium and epithelial basement membrane (EBM) and/or endothelium and Descemet's basement membrane (BM) are commonly disrupted after corneal injuries, surgeries, and infections. Regeneration of these critical regulatory structures relies on the coordinated production of BM components, including laminins, nidogens, perlecan, and collagen type IV by epithelial, endothelial, and keratocyte cells. Whether a cornea, or an area in the cornea, heals with transparency or fibrosis may be determined by whether there is injury to one or both corneal basement membranes (EBM and/or Descemet's BM) and delayed or defective regeneration or replacement of the BM. These opaque myofibroblasts, and the disordered extracellular matrix these cells produce, persist in the stroma until the EBM and/or Descemet's BM is regenerated or replaced.

**Conclusions:**

Corneal stromal fibrosis (also termed “stromal scarring” or “late haze”) occurs as a consequence of BM injury and defective regeneration in both the anterior (EBM) and posterior (Descemet's BM) cornea. The resolution of fibrosis and return of stromal transparency depends on reestablished BM structure and function. It is hypothesized that defective regeneration of the EBM or Descemet's BM allows key profibrotic growth factors, including transforming growth factor beta-1 (TGF-β1) and TGF-β2, to penetrate the stroma at sustained levels necessary to drive the development and maintenance of mature opacity-producing myofibroblasts from myofibroblast precursors cells, and studies suggest that perlecan and collagen type IV are the critical components in EBM and Descemet's BM that bind TGF-β1, TGF-β2, platelet-derived growth factor, and possibly other growth factors, and regulate their bioavailability and function during homeostasis and corneal wound healing.

Scarring of the corneal stroma (Fig. 1), also known as fibrosis or late haze, commonly occurs after injury, infection, or surgery to the cornea involving the epithelium and underlying stroma^1–6^ and/or the endothelium and posterior stroma.^7^ The resultant disorder of stromal transparency and function can, at least in some cases, resolve months to years after the precipitating insult.^3–5^ Clinical factors commonly associated with corneal fibrosis include surgeries such as photorefractive keratectomy (PRK) or the donor-recipient interface of penetrating keratoplasty, traumatic stromal injuries, chemical and thermal burns, microbial infections of the epithelium, stroma and/or endothelium, and delays in corneal epithelial healing after abrasions or therapeutic debridement.^7–11^ Other factors that have been associated with fibrosis include genetic variations, dietary concerns, and environmental influences, such as ultraviolet light exposure.^12^

**Figure 1:**
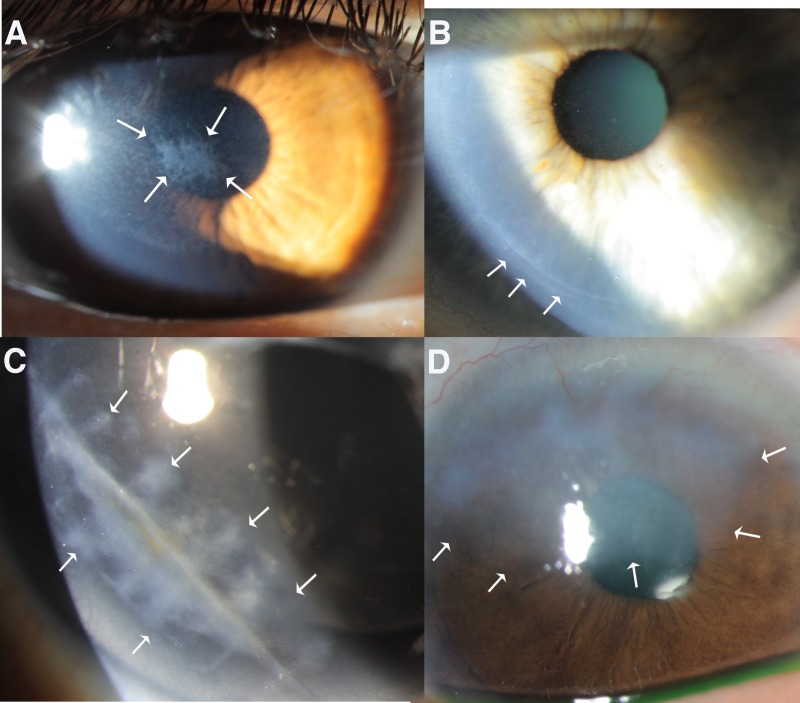
Corneal fibrosis resulting (A) after photorefractive keratectomy (PRK) despite intraoperative mitomycin C treatment (breakthrough haze), (B) at the flap edge after normal laser in situ keratomileusis (LASIK), (C) after a perforating corneal laceration, and (D) following microbial keratitis in human corneas. In all cases, there was delayed regeneration of the epithelial basement membrane (EBM) and the development of myofibroblasts that produce disordered extracellular matrix in the stroma. Arrows indicate scarring.

This review aims to detail the importance of the corneal basement membranes (epithelial basement membrane [EBM] and Descemet's basement membrane [BM]) in the maintenance of corneal transparency and the role of BM injury and defective regeneration after injury, surgery, or infection in the development of myofibroblast-mediated stromal fibrosis. The role of Descemet's BM injury in posterior corneal fibrosis has only recently been recognized^7^ and remains poorly characterized, thus this review will necessarily focus more on the EBM.

## Factors Associated With Corneal Fibrosis

Injuries, surgeries, infections, and diseases that trigger myofibroblast-associated corneal fibrosis have in common disruption of the corneal BMs and delayed regeneration of the BMs.^7,13,14^ Transient haze or mild opacity associated with corneal abrasions, normal PRK,^1–3^ or routine riboflavin-UV cross-linking,^15^ for example, also cause disruption of the EBM, but in these situations the normal regeneration of the EBM halts the development of myofibroblast precursor cells and triggers apoptosis of the myofibroblast precursors and subsequent repopulation of the stroma with keratocytes after termination of the early injury-related wave of keratocyte apoptosis.^7,8,13,14^

PRK and laser in situ keratomileusis (LASIK) provide readily reproducible models for the development of anterior corneal stromal fibrosis. An increased understanding of the factors that promote stromal fibrosis rather than stromal transparency in PRK and LASIK has been of critical importance in understanding fibrosis that occurs after other injuries, surgeries, and infections of the cornea. PRK causes disruptions of the EBM overlying the central cornea, whereas normal LASIK results in disruptions of only the peripheral EBM at the flap edge.^4^ Thus, in normal LASIK surgery, fibrosis is typically noted only at the flap margin (Fig. 1B). However, after some LASIK complications, such as buttonhole flaps or central corneal epithelial sloughs, disruptions of the EBM overlying the central cornea may lead to localized central corneal fibrosis.^16^

**Figure 2:**
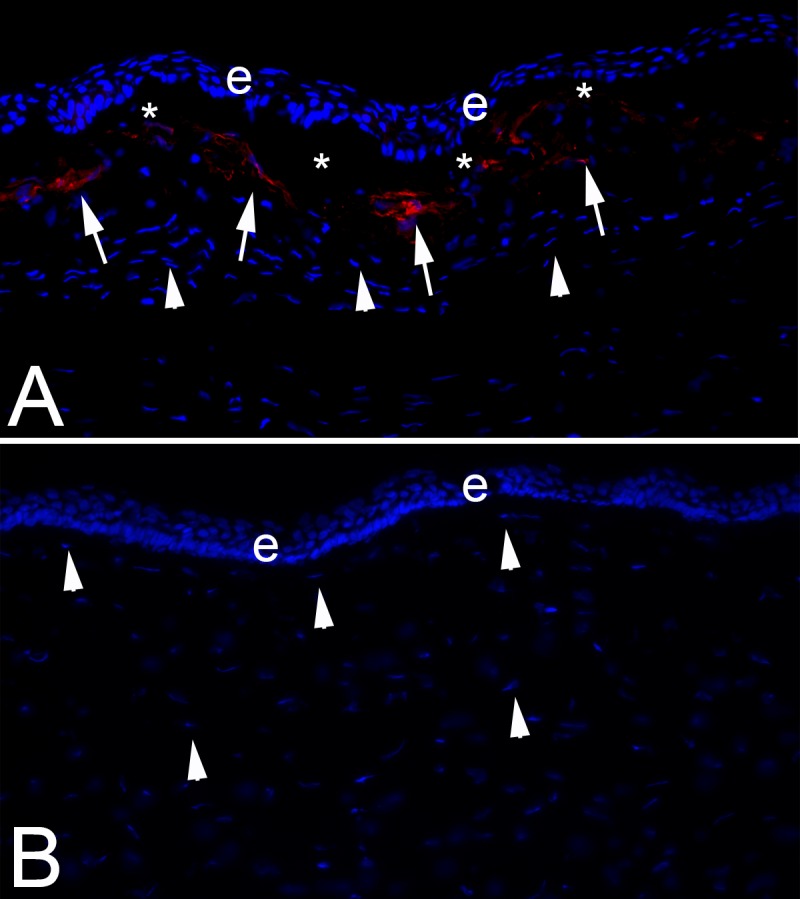
Immunohistochemistry for the alpha-smooth muscle actin (αSMA) marker for myofibroblasts in a rabbit cornea at 1 month after (A) −9 D PRK or (B) −4.5 D PRK. Arrows indicate αSMA^+^ myofibroblasts in the anterior stroma of the cornea that had −9 D PRK immediately beneath the epithelium that is the ongoing source of TGF-β and PDGF that maintains the viability of these fibrotic cells and prevents their apoptosis. Arrowheads indicate keratocytes or corneal fibroblasts that in the −9 D PRK cornea appear at increased density beneath the line of more anterior myofibroblasts. Asterisks in (A) indicate artifactual detachment of the epithelium from the underlying stroma in −9 D PRK corneas that are formed during section cutting with the cryostat since these corneas do not have a normal EBM that adheres epithelium to underlying stroma. Blue is DAPI staining of all cell nuclei. Magnification 400×.

The development of mature myofibroblasts in the corneal stroma, and the persistence of these cells, is the critical stage in the development of fibrosis of the stroma (Fig. 2).^4,17–20^ Driven by TGF-β, platelet-derived growth factor (PDGF), and possibly other unidentified factors, the best-characterized precursors to these cells in corneal stroma (keratocyte-derived and fibrocyte-derived precursors) transition through convergent developmental programs from vimentin^+^, alpha-smooth muscle actin (αSMA)^−^, desmin^−^ (V^+^A^−^D^−^) immature myofibroblasts to vimentin^+^, αSMA^+^, desmin^−^ (V^+^A^+^D^−^) intermediate cells to vimentin^+^, αSMA^+^, desmin^+^ (V^+^A^+^D^+^) mature myofibroblasts.^21^ This developmental maturation process, and the secretion of disordered extracellular matrix once the myofibroblasts are mature, explains why fibrosis typically does not appear until 2 to 4 months after PRK in humans and why corneal fibrotic scars tend to intensify over time after corneal infections, trauma, or surgery. Myofibroblasts are contractile cells that express decreased intracellular crystallins^17^ and that produce disordered extracellular matrix components that alter the normal organization of the corneal stromal collagen lamellae to diminish transparency.^2,4,17^ This contrasts with corneal fibroblasts (also referred to as “activated keratocytes”) that are also generated by injuries, surgeries, infections, or diseases of the cornea. Corneal fibroblasts also show decreased expression of corneal crystallins and an area relatively opaque compared to keratocytes,^17^ but they tend to be transient, produce only small amounts of disordered extracellular matrix, and are generated whether or not there are persistent EBM defects. Thus, transient haze noted after normal PRK, rapidly healing corneal abrasions, or normal riboflavin-UV cross-linking is attributable to corneal fibroblast generation without the production of mature myofibroblasts.^8,13–15^

Myofibroblast development and persistence is dependent on ongoing sufficient concentrations of critical growth factors—especially TGF-β and PDGF.^22–25^ Although stromal cells themselves produce TGF-β, especially after injuries, the amount produced is insufficient to drive myofibroblast generation from either keratocyte-derived or bone marrow–derived precursors.^22,23^ Sufficiently large amounts of TGF-β can, however, be provided by the epithelium (or the aqueous humor in the case of posterior injuries to the cornea,^7,26^ such as lamellar keratoplasty surgery).^27,28^ Clearly, there are mechanisms in place to regulate the penetration of epithelial- and aqueous humor–derived TGF-β, PDGF, and possibly other profibrotic cytokines and growth factors into the stroma to maintain corneal transparency and normal function. The EBM and the corneal EBM (Descemet's BM) are the critical regulatory structures limiting the access of activated TGF-β and PDGF into the stroma via the anterior and posterior corneal surfaces, respectively.^7,13,14^ Thus, normal EBM effectively limits the passage of profibrotic heparin-binding TGF-β1, TGF-β2, and all isoforms of PDGF, but importantly, not antifibrotic, non-heparin-binding TGF-β3.^29,30^ The mechanisms of posterior corneal fibrosis mediated by endothelial and Descemet's BM injury are likely similar to those that occur with anterior corneal fibrosis mediated by epithelial and EBM injury,^7^ but this review will primarily focus on anterior stromal fibrosis since more extensive research has been performed on the latter.

**Figure 3:**
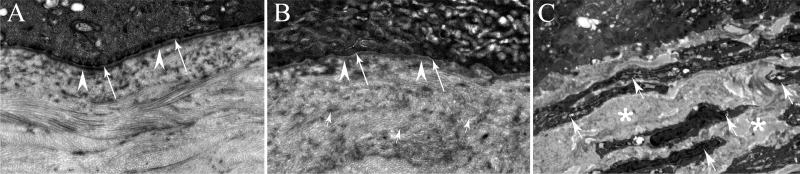
Transmission electron microscopy (TEM) of the corneal EBM and anterior stroma. (A) In a normal cornea, without prior injury or surgery, the normal lamina lucida (arrows) and lamina densa (arrowheads) are noted. (B) At 1 month after −4.5 D PRK in a rabbit, there is normal regeneration of the EBM lamina lucida (arrows) and lamina densa (arrowheads) and there are no detectible myofibroblasts in the anterior stroma. Typically, the fully regenerated EBM can be noted by 8 to 10 days after PRK by TEM.^34^ Note that collagen lamellae of normal diameter and packing can be seen throughout the anterior stroma (small arrows). (C) At 1 month after −9 D PRK in a rabbit cornea, there is no detectible lamina lucida or lamina densa. In addition, the anterior stroma is filled with stacks of cells (arrows) with prominent endoplasmic reticulum that correspond to the αSMA^+^ myofibroblasts in Figure 2A and disordered extracellular matrix secreted by the myofibroblasts (asterisks). Magnification 23,000×.

At sites of EBM injury, TGF-β and PDGF released from epithelial cells and secreted into tears from the conjunctiva and lacrimal gland penetrate into the stroma and initiate the development of myofibroblasts from precursors.^21,24,31^ After most superficial injuries to the cornea, where the epithelium heals in a timely manner, such as abrasions, riboflavin-UV cross-linking, and normal PRK, the EBM is regenerated within 8 to 10 days (Fig. 3).^32^ Subsequently, myofibroblast precursors that have begun development are deprived of the requisite supply of TGF-β and PDGF and undergo apoptosis before they become established in the anterior stroma and develop into mature myofibroblasts that secrete large amounts of disordered extracellular matrix.^13,14,33^ Conversely, if the epithelium doesn't heal in a timely manner or there is defective regeneration of the underlying EBM (Fig. 3), then TGF-β and PDGF, and possibly other profibrotic growth factors, continue to penetrate into the stroma at sufficient levels to drive the development and persistence of mature subepithelial myofibroblasts (Fig. 3).

Studies in chimeric mice expressing green fluorescent protein only in their bone marrow–derived cells have conclusively demonstrated that corneal stromal myofibroblasts arise from both keratocyte-derived and bone marrow–derived fibrocyte precursor cells.^31,34,35^ In an individual mouse cornea that develops anterior stromal haze after irregular PTK, 30% to 70% of myofibroblasts are derived from bone marrow–derived precursors, with the remainder being derived from keratocytes.^24^ It is unknown why there are multiple sources of progenitors to myofibroblasts in cornea, or other organs that have been studied,^36^ including under some circumstances Schwann cells in the cornea.^37^ However, in vitro mouse coculture experiments demonstrated that the percentage of αSMA^+^ myofibroblasts generated in culture was significantly higher in the presence of TGF-β when bone marrow–derived cells and corneal stromal fibroblasts were cocultured together compared to when bone marrow–derived cells or corneal stromal fibroblasts were cultured individually.^36^ Thus, multiple precursor cells, and possibly their progeny, may interact to enhance the fibrotic response in the tissue after severe injuries.

## Defective EBM Regeneration

Thus, defective EBM regeneration is a critical factor leading to myofibroblast generation and persistence and resulting anterior corneal stromal fibrosis.^13,14,38^ But what causes the EBM to not regenerate normally and anterior stromal fibrosis to develop after a −9 diopter (D) PRK in one individual when the EBM regenerates normally with maintenance of stromal transparency in another individual with the exact same PRK procedure? Thus, even in the absence of mitomycin C treatment, the incidence of stromal fibrosis after PRK corrections greater than 5 to 6 D of myopia in humans is only approximately 2% to 6% of eyes.^1–3^ Similarly, why does the EBM nearly always regenerate normally and the stroma remain transparent after PRK for low myopia when abnormal EBM regeneration and stromal fibrosis occur commonly after the same exact PRK procedure performed for higher myopia?^1–3^ Finally, why was corneal fibrosis (late haze) found to be much more common when PRK was performed with some excimer lasers (for example, the Summit excimer laser; Summit Technology, Inc., Waltham, MA, USA; or the Autonomous excimer laser; Autonomous Technologies, Corp., Orlando, FL, USA) than with other brands of excimer lasers (for example, the VISX excimer laser; VISX, Inc., Santa Clara, CA, USA) prior to the routine use of mitomycin C in PRK?^14^

The first factor to be appreciated was the mechanical effect of stromal surface irregularity retarding EBM regeneration (Vinciguerra and Epstein, unpublished observations, 2001) made the clinical observation that including a corneal surface-smoothing PRK treatment after PRK for high myopia decreased the incidence of late haze (anterior stromal fibrosis) in human corneas. Subsequently, using a rabbit PRK model, Netto and colleagues^20^ demonstrated that there was a direct correlation between the level of surface irregularity after PRK and (1) the myofibroblast density in the anterior stroma and (2) the severity of stromal opacity. Thus, lasers such as the Summit excimer laser or Autonomous excimer laser, which have been shown to produce greater surface irregularity during stromal ablation,^39^ also trigger greater anterior stromal fibrosis than lasers such as the VISX STAR S4/IR (Abbott Medical Optics, Inc., Santa Ana, CA, USA) or the WaveLight Allegretto Wave Eye-Q (Alcon Laboratories, Inc., Fort Worth, TX, USA) excimer lasers that generate smoother stromal surfaces.^40^

There are, however, other factors besides stromal surface irregularity that can lead to defective EBM regeneration since corneas that have PTK smoothing after PRK may still develop anterior stromal fibrosis without postoperative mitomycin C treatment. Another factor involves important cellular interactions involved in EBM regeneration after injury. Many studies in the cornea^41–43^ and other organs^44–51^ have demonstrated that fibroblastic or mesenchymal cells produce BM components such as laminins, nidogens, and perlecan that contribute to the development or regeneration of BMs needed to maintain normal tissue morphology and function. In a recent study, Santhanam and coworkers^32^ used laser capture microdissection-quantitative reverse transcriptase polymerase chain reaction to study EBM component mRNA production in the anterior stroma of rabbit corneas after −4.5 D PRK or −9 D PRK. The authors found that the messenger RNAs for EBM components nidogen-2 and laminin alpha-3 (LAMA3), but not nidogen-1 or perlecan, were significantly reduced in the subepithelial anterior stroma of the −9 D PRK corneas at 4 and 7 days after surgery—the time interval just prior to ultrastructural appearance of normal lamina lucida and lamina densa in −4.5 D PRK corneas (8 to 10 days after surgery) that did not develop fibrosis.^32^ Torricelli and coworkers^33^ showed that rabbit corneas that develop anterior fibrosis, or late haze, after PRK had no detectible EBM lamina lucida and lamina densa when the ultrastructure was examined with transmission electron microscopy (TEM).

Nidogens and perlecan have critical roles in linking other BM components, acting as bridging molecules between components.^33,45^ Thus, nidogens have a strong affinity for perlecan, laminins, and collagen type IV,^45^ and deficiencies in nidogens in skin-organotypic coculture models lead to defective EBM assembly in vitro.^52^

What components in the EBM and Descemet's BM are likely involved in binding TGF-β1, TGF-β2, PDGF, and other cytokines and growth factors to modulate their bioavailability in the stroma and therefore corneal fibrosis? All TGF-β family members, including TGF-β1 and TGF-β2, are synthesized as precursor molecules with a N-terminal prodomain, C-terminal mature domain and a signal peptide for extracellular transport.^53^ Dimeric TGF-β family members are excreted from the cell of origin and interact with extracellular matrix proteins, including perlecan, to regulate their bioavailability and function.^53^ Thus, perlecan in EBM and Descemet's BM likely has a critical role in modulating the function of TGF-β1 and TGF-β2 excreted by corneal epithelial cells or endothelial cells, or is present in the aqueous humor. Similarly, TGF-β1 and TGF-β2 bind to collagen type IV, a major component of EBM and Descemet's BM.^54^ Finally, both PDGF-AA and PDGF-BB bind to perlecan, collagen type IV, and nidogen present in EBM and Descemet's BM.^55^ Therefore, at least three components of EBM and Descemet's BM likely bind TGF-β1, TGF-β2, and PDGF and thereby control their bioavailability to stimulate the development of myofibroblasts from precursor cells. We hypothesize that when no EBM or Descemet's BM is present, or when these BMs are defective, TGF-β1, TGF-β2, and PDGF from corneal epithelial cells, endothelial cells, or aqueous humor penetrate the stroma at sufficiently high levels to drive development of myofibroblasts and maintain their viability. BM components have similarly been shown to regulate the bioavailability of other growth factors and cytokines.^56–58^ It is important to point out that this regulatory role of the EBM is likely bidirectional since keratinocyte growth factor (KGF or FGF-7) produced by keratocytes and corneal fibroblasts to regulate the proliferation, motility, and differentiation of corneal epithelial cells^59,60^ also specifically bind to the perlecan protein core.^61^

The working hypothesis is that once the self-assembling nascent laminin-511 and/or -521 layer is laid down beneath basal epithelial cells,^62^ followed by addition of laminin-332, this layer creates a barrier to penetration of more posterior EBM components from the epithelial cells, and at least some of these EBM components (including possibly nidogens, perlecan, laminins, and collagens) must be provided by keratocytes in the cornea. Thus, if there are insufficient keratocytes in the anterior stroma in proximity to the nascent EBM, then full regeneration of the mature EBM is delayed and myofibroblast precursors are driven by TGF-β and PDGF (that, therefore, penetrate into the stroma) to develop into mature myofibroblasts.

In the corneal laser microdissection study of EBM component expression,^32^ the authors hypothesized that increased anterior stromal keratocyte apoptosis that occurs immediately after high correction −9 D PRK compared to low correction −4.5 D PRK^8^ contributes to decreased anterior stromal keratocytes and the EBM components they produce in the early postoperative period when the EBM normally regenerates. Differences were also noted in the intracellular localization of EBM component proteins, perhaps related to secretion, in keratocytes and myofibroblasts cultured in vitro.^43^ Thus, it was hypothesized that keratocytes must be in proximity to the nascent EBM during regeneration for a fully functional, mature EBM to be regenerated by active, coordinated function of the epithelium and the keratocytes.^32^ It is likely that studies that found correlations between the stromal volume ablated by PRK and the incidence and severity of stromal haze^63^ were also detecting a correlation between the availability of keratocytes in the anterior stroma to facilitate regeneration of the EBM.

A clinical corollary to the critical participation of keratocytes in regeneration of the injured EBM is that surgeons should strive to preserve as many keratocytes as possible during surgical procedures such as PRK and riboflavin-UV cross-linking. Many surgeons utilize ethanol to facilitate the removal of the epithelium during these surgeries and treatments. However, use of ethanol significantly increases the death of stromal keratocytes beyond mechanical epithelial removal alone.^64^ Thus, surgeons who unnecessarily use ethanol in these procedures may unwittingly increase the risk of anterior stromal fibrosis (late haze) after PRK, even during PRK surgery for lower corrections of myopia or when mitomycin C is used to reduce fibrosis (“breakthrough haze” after PRK; Fig. 1A). Similarly, removal of more epithelium and EBM than necessary for the diameter of the excimer laser PRK or the use of rotary brushes to remove epithelium (that more efficiently break open epithelial cells and release more proapoptotic cytokines) may augment the early stromal keratocyte apoptosis response^8,65^ and reduce the overall population of keratocytes in the anterior corneal stroma during the critical early phase of epithelial healing and EBM regeneration when these keratocytes coordinate with the overlying epithelium in the restoration of normal corneal EBM structure and function. Surgeons who experience an unusually high incidence of late haze or breakthrough late haze (Fig. 1A) after PRK, even after lower corrections for myopia, should carefully review their techniques to ascertain whether they are inadvertently augmenting the early keratocyte apoptosis response to the detriment of the EBM regeneration process.

Clinical studies have suggested a correlation between ultraviolet light exposure and the development of corneal fibrosis (late haze) after PRK.^12^ The pathophysiology of this correlation remains uncertain.

There are likely genetic factors that influence the regeneration of the EBM and the development of anterior stromal fibrosis after PRK and other injuries, infections, and treatments. Thus, patients who develop fibrosis (late haze) in the cornea of one eye after PRK typically develop similar fibrosis in the opposite eye, although unilateral cases have been described.^9^ Also, rare patients who have PRK for corrections as low as 1 D of myopia develop bilateral anterior stromal fibrosis (late haze).^14^ Specific genetic factors involved in the development of anterior stromal fibrosis have not been reported. However, subtle genetic anomalies in EBM components or other genes involved in the mechanics of BM regeneration or repair are likely candidates for study.

Finally, corneas that have had prior PRK or other corneal surgeries (such as LASIK) are at increased risk of developing anterior stromal fibrosis after retreatment with PRK, even years after the original surgery—especially if mitomycin C treatment is not included at the time of the retreatment. Repeat PRK or PRK enhancement following LASIK could result in a greater decrease in anterior stromal keratocytes available to participate in the EBM regeneration process.^32^ Prior LASIK surgery provides a potential space beneath the original LASIK flap that facilitates penetration of bone marrow–derived myofibroblast precursor cells from limbal blood vessels into the central subepithelial stroma that promotes the development of corneal fibrosis analogous to the increased risk of diffuse lamellar keratitis following corneal abrasion months to years after LASIK.^66^

**Figure 4:**
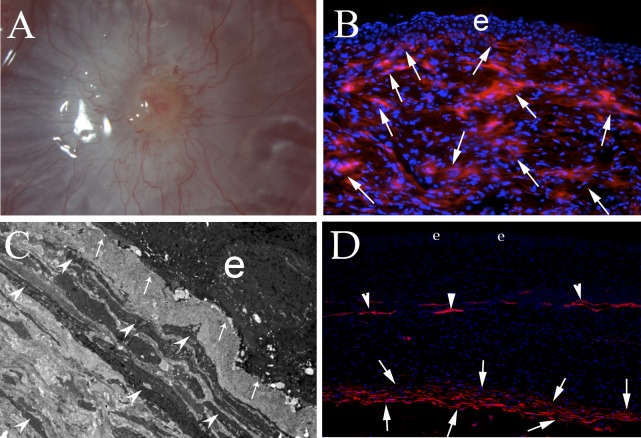
Fibrosis after bacterial keratitis. (A) Slit lamp photo of a fibrotic rabbit cornea 1 month after Pseudomonas aeruginosa keratitis treated at 24 hours after infection with topical tobramycin. Magnification 40×. (B) Immunohistochemistry for the αSMA marker for myofibroblasts at 1 month after P. aeruginosa keratitis showing the stroma packed with red-stained myofibroblasts (arrows). e, epithelium. Magnification 200×. (C) TEM of anterior stroma 1 month after P. aeruginosa keratitis and treatment showing no detectible BM lamina lucida or lamina densa (arrows) and the anterior stroma filled with layers of myofibroblasts (arrowheads) corresponding to the αSMA^+^ cells in (B). e, epithelium. Magnification 23,000×. (D) At 3 months after P. aeruginosa infection and treatment, immunohistochemistry for αSMA shows that myofibroblasts have mostly disappeared beneath the intact epithelium (e) and regenerated EBM (not shown, but see full study^8^) from the anterior to deep stroma beneath the regenerated EBM. αSMA^+^ pericytes remain associated with neovascular blood vessels (arrowheads). Beneath the posterior surface of the stroma, however, there persists a layer of αSMA+ myofibroblasts (arrows) where Descemet's BM and the corneal endothelium were destroyed by the original infection and did not regenerate. The TGF-β requisite to maintain the viability of these myofibroblasts likely enters the stroma from the aqueous humor^76^ but only penetrates the stroma at the necessary concentration for a small distance. Magnification 100×.

The factors that have been discovered to underlie the development of corneal fibrosis after PRK are likely also relevant to corneal injuries, infections, and burns, although few studies have been performed to explore the pathophysiology of corneal fibrosis in these other disorders. However, a recent study of *Pseudomonas aeruginosa* keratitis in rabbits^8^ demonstrated that defective regeneration of the EBM and Descemet's BM is associated with anterior and posterior corneal fibrosis, respectively (Fig. 4).

Opacity and fibrosis often persist for a period of time measured in years or decades following incisional wounds such as corneal lacerations, radial keratotomy, or astigmatic keratotomy. It is known that EBM is often regenerated around epithelial plugs that often extend into the corneal stroma after incisional injuries.^38^ Presumably, however, this ectopic EBM does not become mature in some cases, and myofibroblasts persist due to continued availability of TGF-β. Further research into fibrosis associated with these incisional wounds is needed.

## Spontaneous Resolution of Corneal Fibrosis

**Figure 5:**
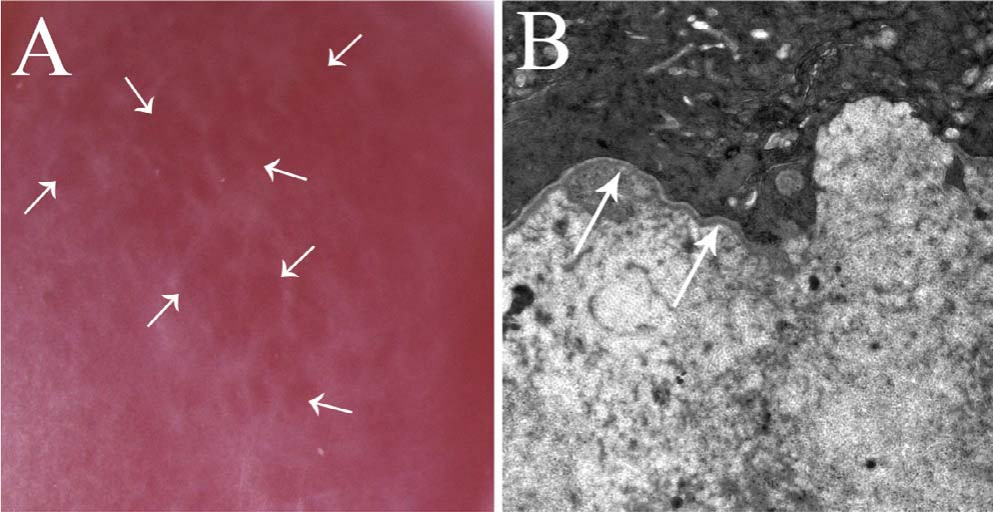
Lacunae in fibrosis (late haze) after PRK in a rabbit cornea. (A) Slit lamp photo of clearer lacunae (arrows) in rabbit cornea with fibrosis at 2 months after −9 D PRK. Magnification 40×. (B) TEM of an area with lacunae and regeneration of lamina lucida and lamina densa (arrows) adjacent to an area on the right with no detectible EBM. Magnification 23,000×.

Corneal fibrosis may undergo spontaneous resolution over months to years with a return of partial or full corneal transparency.^13,14,67,68^ A recent study of PRK in rabbits demonstrated that this occurs via gradual restoration of EBM ultrastructural morphology and function.^38^ When the resolution begins, areas of clearing referred to as “lacunae” appear in a spotty distribution within the fibrotic opacity (Fig. 5).^38^ If these lacunae are examined with TEM, it is noted that normal EBM ultrastructure with lamina lucida and lamina densa has been restored in these islands within the stroma and myofibroblasts have disappeared, but not in the adjacent opaque cornea with fibrosis that remains. Once sufficient EBM is regenerated, other myofibroblasts in adjacent areas underlying remaining defects in EBM disappear—presumably because the stromal concentrations of TGF-β and PDGF have fallen below a critical level needed for myofibroblast viability. We hypothesize that these lacunae represent locations where keratocytes have managed to penetrate through the fibrotic band of myofibroblasts, perhaps by inducing paracrine IL-1α–mediated myofibroblast apoptosis,^25,68^ and then cooperate with overlying epithelial cells to restore EBM structure and function. Once the myofibroblasts die, more keratocytes repopulate this stroma and reabsorb disordered extracellular matrix to restore transparency, causing the lacunae to enlarge and coalesce to restore full transparency of the entire PRK-ablated zone.^38^ In other cases, the process is incomplete, and clear areas remain intermixed with opaque fibrotic areas without apparent change for many years. Unfortunately, in some corneas, the clearing of opacity remains inadequate to restore functional vision, presumably because the EBM was never fully regenerated, and phototherapeutic keratectomy, lamellar keratoplasty, or penetrating keratoplasty becomes necessary.^14^

## Mitomycin C Treatment and Corneal Fibrosis

**Figure 6:**
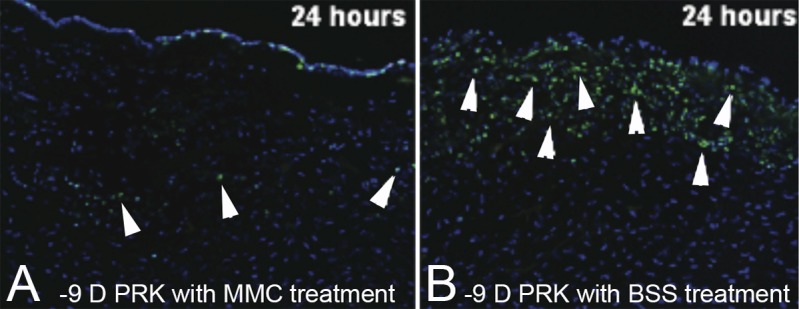
Effect of mitomycin C (MMC) treatment on stromal cell mitosis after −9 D PRK. Immunohistochemistry for mitosis marker Ki-67 at (A) 24 hours after −9 D PRK followed by treatment with 0.02% MMC for 2 minutes and (B) 24 hours after −9 D PRK followed by treatment with vehicle control balanced salt solution (BSS) for 2 minutes. Notice after MMC treatment there are far fewer anterior stromal cells undergoing mitosis (arrowheads in [A] than there are after BSS treatment (arrowheads in [B]). This MMC blockade of anterior stromal cell mitosis continued for over 1 month after PRK and inhibited proliferation of the precursor cells that would have developed into mature myofibroblasts associated with corneal fibrosis or late haze. Reprinted from Netto MV, Mohan RR, Sinha S, Sharma A, Gupta PC, Wilson SE. Effect of prophylactic and therapeutic mitomycin C on corneal apoptosis, cellular proliferation, haze, and long-term keratocyte density in rabbits. J Refract Surg. 2006;22:562–574, with permission from SLACK Incorporated.

How does mitomycin C treatment markedly reduce the incidence of anterior stromal fibrosis or late haze after PRK? Netto et al.^69^ demonstrated that the most important effect of mitomycin C is to block TGF-β and PDGF-driven mitosis of stromal cells (Fig. 6), including myofibroblast precursor cells, during the critical phase of myofibroblast development when mitosis of either keratocyte-derived or bone marrow–derived precursors must occur to produce clinically significant anterior stromal fibrosis. Thus, despite there being EBM defects in some corneas after PRK for high myopia, treatment with mitomycin C usually inhibits myofibroblast progenitors from proliferating to sufficient numbers to establish the subepithelial layer of myofibroblasts (Fig. 2B and Fig. 3C) that causes fibrosis and prevents keratocytes from repopulating the anterior stroma.

Mitomycin C treatment isn't always effective in blocking anterior stromal fibrosis after PRK, and opacity that occurs after PRK despite mitomycin C treatment is referred to as “breakthrough haze.” When breakthrough haze occurs (Fig. 1A),^70^ there is much less chance that the anterior stromal fibrosis will resolve spontaneously, even years after the PRK surgery.^13,70^ Presumably, this is due to long-term effects of mitomycin C on anterior stromal repopulation with keratocytes, persistent keratocyte phenotypic changes, or other unknown factors.^70^

## Treatment of Corneal Fibrosis

Since corneal fibrosis often spontaneously resolves, the best course of action after surgery, trauma, or infection complicated by clinically significant haze is to wait for a period of at least a year to observe whether lacunae (Fig. 5A) begin to appear within the opacity.^14,38^ If this fibrosis occurred after PRK, patients often have surprisingly good vision with spectacle correction and a refractive error similar to that present in the preoperative eye. If the fibrosis resolves over time, the intended surgical correction of refractive error often reappears—in some cases to the full correction that was planned for the PRK surgery. Thus, patience is a virtue in the management of late haze fibrosis after PRK.

If the fibrosis does not begin to clear by 1 to 2 years, it is not likely to resolve.^14^ Some of these cases respond to PTK with mitomycin C without recurrence of fibrosis or with only partial recurrence and improved function.^71^ However, even with mitomycin C treatment, there is a possibility of worsening fibrosis after PTK treatment. In such cases, penetrating keratoplasty or lamellar keratoplasty may be needed to restore vision.

## Posterior Corneal Fibrosis

**Figure 7:**
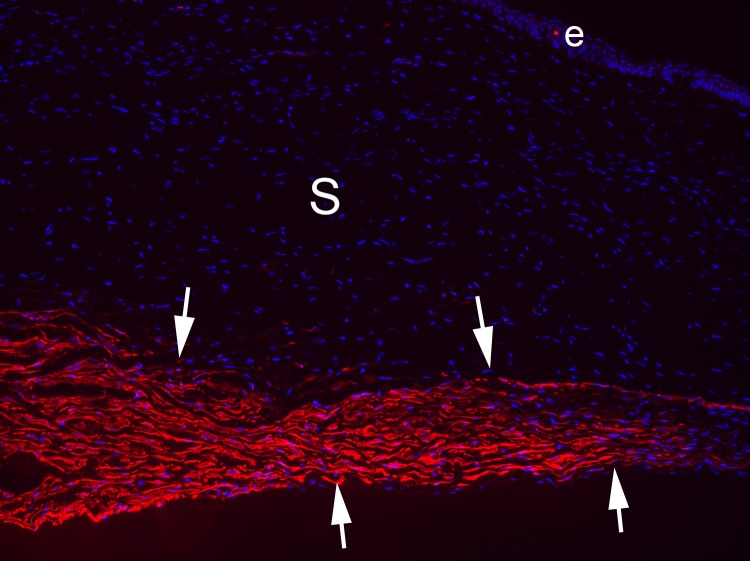
Posterior myofibroblast-mediated corneal fibrosis in the rabbit 1 month after excision of an 8-mm-diameter circle of endothelium and Descemet's BM. The arrows indicate αSMA^+^ myofibroblast cells. e, epithelium; s, stroma. Magnification 100×.

Fibrosis of posterior cornea can occur after infections such as severe microbial infections,^7^ herpes simplex keratouveitis,^72^ diseases such as advanced bullous keratopathy or Fuchs' dystrophy,^73^ or after endothelial replacement surgeries such as penetrating keratoplasty, Descemet's stripping automated endothelial keratoplasty (DSAEK), and Descemet's membrane endothelial keratoplasty (DMEK).^27,74^ One study of DSAEK in cats did not find myofibroblasts associated with the graft-host interface,^75^ but this study did not study sufficiently late time points because αSMA^+^ myofibroblasts would not be expected to develop by 9 days after the surgical injury—the latest time point examined by the investigators. Recent experiments on fibrosis after microbial keratitis demonstrated that the important role of the EBM in modulating fibrosis in the anterior stroma is paralleled by the role of Descemet's BM in the modulation of fibrosis in the posterior stroma.^7^ Thus, when Descemet's BM and the endothelium was damaged in the setting of severe *Pseudomonas aeruginosa* keratitis, posterior corneal myofibroblasts and fibrosis persisted in the stroma, even after the EBM regenerated and myofibroblasts disappeared in the anterior to midstroma (Fig. 4C). The source of the TGF-β required to maintain myofibroblast viability in the posterior stroma was likely the aqueous humor.^76^ In recent studies of this posterior fibrosis response (Medeiros C, Wilson SE, unpublished studies, 2017), it was found that when an 8-mm diameter circle of Descemet's BM and endothelium was excised without replacement, nearly 100% of the corneas developed myofibroblast-mediated posterior fibrosis (Fig. 7), although the level of fibrosis varied between different corneas. Posterior fibrosis does not occur if the endothelium alone is removed over an 8-mm-diameter circle using an olive tip cannula, despite apoptosis of the overlying posterior stromal keratocytes and the ensuing stromal wound healing response.^77^ This experiment confirms that Descemet's BM is the critical modulator of posterior corneal fibrosis, as the EBM is for anterior corneal fibrosis. Further study is needed to fully understand the role Descemet's BM plays in the modulation of posterior corneal fibrosis that occurs after corneal infections, diseases, trauma, and surgeries.

## Conclusions

The corneal BMs are likely critical modulators of the corneal fibrosis response. Following injury, if the EBM is not fully regenerated and its function restored, then epithelium-generated TGF-β and PDGF persistently penetrate into the stroma at sufficient levels to drive the development of myofibroblasts from precursor cells. If TGF-β and PDGF continue to penetrate into the stroma, these profibrotic cells persist indefinitely. Once established, the opaque myofibroblasts produce disordered extracellular matrix that generates fibrosis and reduced stromal transparency. Myofibroblasts also block keratocytes from repopulating the fibrotic area of the stroma. Fibrosis persists until normal EBM is regenerated, likely by the coordinated response of keratocytes that finally penetrate through the fibrotic band to cooperate with epithelial cells in restoring the EBM structure and function in localized areas within the fibrosis. Deprived of adequate levels of epithelial TGF-β and PDGF, underlying myofibroblasts undergo apoptosis and repopulating keratocytes produce lacunae of more transparent anterior stroma. These zones progressively enlarge and coalesce as more keratocytes invade, contribute to regeneration of normal EBM, and reabsorb disordered extracellular matrix produced by the myofibroblasts. Descemet's BM serves a parallel function in the posterior cornea, and damage to Descemet's BM can lead to the development and/or persistence of posterior myofibroblasts and fibrosis. The association of BM injury and defective regeneration with myofibroblast-mediated fibrosis may be relevant to fibrosis in other organs such as lung, liver, kidney, heart, and skin.^68^

## Supplementary Material

Supplement 1
