# Supplementary material for: The Corneal Basement Membranes and Stromal Fibrosis
Source: Invest Ophthalmol Vis Sci. 2018 Aug;59(10):4044–53. doi: 10.1167/iovs.18-24428 (PMC6088801; doi:10.1167/iovs.18-24428)
Supplement: Supplement 1 [file iovs-59-10-11_s01.pdf]

SLACK
INCORPORATED

April 5, 2018

Steven Wilson
Cole Eye Institute, The Cleveland Clinic
9500 Euclid Ave, Cleveland OH 44195

Reference #: J21736361
Material Requested: Two of the panels in Figure 6 (top left and top right)
Usage Requested: Used in a review article titled "The Corneal Basement Membranes Modulate Stromal Fibrosis" to be submitted for publication in Investigative Ophthalmology and Visual Sciences, published by the Association for Research in Vision and Opthalmology.
Citation: Netto M, Mohan R, Sinha S, Sharma A, Gupta P, Wilson S. Effect of Prophylactic and Therapeutic Mitomycin C on Corneal Apoptosis, Cellular Proliferation, Haze, and Long-term Keratocyte Density in Rabbits. J Refract Surg. 2006; 22: 562-574

Dear Steven,

Permission is granted for the requested materials and usage listed above, subject to the following conditions:

- Permission is granted for **one-time use only**. Permission does not apply to future editions, revisions, or derivative works.
- Permission is granted for non-exclusive, worldwide use, in the English language, in print and electronic form. Requests for additional formats, languages, or future editions must be submitted separately.
- At no time may the materials appear on a general website and must appear **only** on a password-protected site.
- The material (eg, figure image, table) requested will not be provided by SLACK Incorporated.
- The following credit line must be displayed: CITATION. Reprinted with permission from SLACK Incorporated. See above for citation information.
- The fee for this use is **$0.00 USD**. This offer is valid for 180 days from the date on this letter. If the requestor does not sign, return, and issue payment during this period, then the permission is rescinded.

Please sign and date below, keep a copy for your records, and fax to Attn: Permissions Department. Please include your reference number on all correspondence and payment information. A copy of this form **MUST** accompany payment.

Requestor accepts conditions above:

Signature: [signature] M.D. **Date:** 4-5-2018

Sincerely,
SLACK Incorporated
Permissions Department
